# Knowledge barriers in a national symptomatic-COVID-19 testing programme

**DOI:** 10.1371/journal.pgph.0000028

**Published:** 2022-01-19

**Authors:** Mark S. Graham, Anna May, Thomas Varsavsky, Carole H. Sudre, Benjamin Murray, Kerstin Kläser, Michela Antonelli, Liane S. Canas, Erika Molteni, Marc Modat, M. Jorge Cardoso, David A. Drew, Long H. Nguyen, Benjamin Rader, Christina Hu, Joan Capdevila, Alexander Hammers, Andrew T. Chan, Jonathan Wolf, John S. Brownstein, Tim D. Spector, Sebastien Ourselin, Claire J. Steves, Christina M. Astley

**Affiliations:** 1 School of Biomedical Engineering & Imaging Sciences, King’s College London, London, United Kingdom; 2 Zoe Global Limited, London, United Kingdom; 3 Department of Population Science and Experimental Medicine, MRC Unit for Lifelong Health and Ageing, University College London, London, United Kingdom; 4 Department of Computer Science, Centre for Medical Image Computing, University College London, London, United Kingdom; 5 Clinical and Translational Epidemiology Unit, Massachusetts General Hospital and Harvard Medical School, Boston, MA, United States of America; 6 Computational Epidemiology Lab, Boston Children’s Hospital, Boston, MA, United States of America; 7 Department of Epidemiology, Boston University School of Public Health, Boston, MA, United States of America; 8 King’s College London & Guy’s and St Thomas’ PET Centre, School of Biomedical Engineering and Imaging Sciences, London, United Kingdom; 9 Department of Twin Research and Genetic Epidemiology, King’s College London, London, United Kingdom; 10 Division of Endocrinology, Boston Children’s Hospital, Boston, MA, United States of America; 11 Broad Institute of Harvard and MIT, Cambridge, MA, United States of America; Instituto Nacional de Geriatria, MEXICO

## Abstract

Symptomatic testing programmes are crucial to the COVID-19 pandemic response. We sought to examine United Kingdom (UK) testing rates amongst individuals with test-qualifying symptoms, and factors associated with not testing. We analysed a cohort of untested symptomatic app users (N = 1,237), nested in the Zoe COVID Symptom Study (Zoe, N = 4,394,948); and symptomatic respondents who wanted, but did not have a test (N = 1,956), drawn from a University of Maryland survey administered to Facebook users (The Global COVID-19 Trends and Impact Survey [CTIS], N = 775,746). The proportion tested among individuals with incident test-qualifying symptoms rose from ~20% to ~75% from April to December 2020 in Zoe. Testing was lower with one vs more symptoms (72.9% vs 84.6% p<0.001), or short vs long symptom duration (69.9% vs 85.4% p<0.001). 40.4% of survey respondents did not identify all three test-qualifying symptoms. Symptom identification decreased for every decade older (OR = 0.908 [95% CI 0.883–0.933]). Amongst symptomatic UMD-CTIS respondents who wanted but did not have a test, not knowing where to go was the most cited factor (32.4%); this increased for each decade older (OR = 1.207 [1.129–1.292]) and for every 4-years fewer in education (OR = 0.685 [0.599–0.783]). Despite current UK messaging on COVID-19 testing, there is a knowledge gap about when and where to test, and this may be contributing to the ~25% testing gap. Risk factors, including older age and less education, highlight potential opportunities to tailor public health messages. The testing gap may be ever larger in countries that do not have extensive, free testing, as the UK does.

## Introduction

Testing is a crucial component of the COVID-19 public health response to guide mitigation and triage illness, even as countries roll out vaccination campaigns. Whilst mass, population-based testing has been trialled [1–3], the majority of programmes seek to test individuals experiencing a certain set of symptoms. A successful program needs high testing uptake among those with test-qualifying symptoms [4]. Achieving high uptake requires both an informed and willing population, and sufficient infrastructure to ensure test availability and accessibility.

In the United Kingdom (UK), the test-qualifying symptoms (fever, cough, or loss of smell) [5] are relatively straightforward, have been consistent since loss of smell was added to the criteria on 18 May 2020, and are buttressed by a free, high-capacity, national testing programme. This is in contrast to other countries where criteria for testing have been more nuanced, varied over time and between regions, and testing access remains suboptimal [6, 7]. Yet, despite the strengths of the UK programme, we observed that 25% of symptom-tracking app participants do not report testing despite experiencing test-qualifying symptoms. This and other evidence [8, 9] raised questions regarding how the path from symptoms to testing could be improved to fully support the pandemic response.

Prior research focuses on logistical barriers for not getting tested such as geographic, socioeconomic and structural disparities in testing access [6, 8, 10], but there are other important barriers, including the knowledge required to successfully navigate the journey from symptom onset to test completion. Examining the reasons why people do not complete testing is hindered by the difficulty in identifying individuals who should have, but did not, receive COVID-19 tests.

Towards this end, we leveraged longitudinal data from over 4 million Zoe COVID Symptom Study (Zoe) participants [11], and over 700,000 surveys from the University of Maryland Global COVID-19 Trends and Impact Survey (UMD-CTIS) [12], to describe the temporal changes in COVID-19 testing among UK residents with test-qualifying symptoms. We followed-up with cross-sectional surveys of the untested, and identified two common barriers to testing: limited knowledge of test-qualifying symptoms as an early barrier and limited knowledge of where to test as a later barrier.

## Materials and methods

This research combines syndromic surveillance data from the UK Zoe COVID Symptom Study (Zoe) [11] and UMD-CTIS [12]. Additionally, more detailed follow-up surveys of recently untested symptomatic participants were analyzed. Survey details provided in Table A-C in S1 Text. Throughout we define test-qualifying symptoms using the UK’s National Health Service (NHS) criteria: high temperature; new, continuous cough; or loss or change to sense of smell or taste [5].

### Data sources

#### UK Zoe COVID Symptom Study (Zoe)

Longitudinal data were prospectively collected using the Zoe COVID Symptom Study app, developed by Zoe Global with input from King’s College London (UK), the Massachusetts General Hospital (Boston, USA), and Lund and Uppsala Universities (Sweden). We used data from app launch on 24 March 2020 through 1 January 2021 (N = 4,394,948, n = 245,505,763 user-reports). App details are published elsewhere [11]. Briefly, participants are asked enrollment questions at baseline, and then daily whether they feel physically normal or if they are experiencing symptoms. Participants are asked to record all COVID-19 test dates, types, and outcomes. To support COVID-19 incidence estimation [13], from 28 April 2020 the UK Department of Health and Social Care (DHSC) allocated polymerase chain reaction (PCR) tests to participants reporting any symptom after ≥1 “well” report in 9 days.

#### UK Zoe follow-up survey

To better understand the reasons why individuals who experience test-qualifying symptoms do not get tested, we deployed a cross-sectional SurveyMonkey web survey. We targeted participants reporting ≥1 test-qualifying symptoms for the first time between 14 November and 8 December 2020, who did not have a COVID-19 swab test report -7 to +14 days from symptom onset, including data entered up through 15 December 2020. Survey responses were linked to Zoe accounts using a unique, anonymised, user identifier. There were four survey sections to assess several testing barriers of interest, including test-qualifying symptom recall and recognition, and test seeking and access. The survey was refined based on analysis of N = 194 pilot survey responses sent to N = 1,000. On 18 December 2020, the final survey was delivered by email to eligible participants (N = 4,936 less N = 706 without valid email address).

#### UK University of Maryland Global COVID-19 Trends and Impact Survey (UMD-CTIS)

This research is based on UK survey results from the University of Maryland (UMD) Social Data Science Center Global COVID-19 Trends and Impact Survey (UMD-CTIS) in partnership with Facebook [12]. UMD delivered web-based, cross-sectional surveys to users sampled from the Facebook active user base. Survey sampling strategies were used to increase representativeness of the source population (here, the UK population) by sampling from the UK Facebook active user base and raking across census age, sex and geographic region to develop survey weights [14]. The study was drawn from N = 775,746 responses within UK geographic regions from 30 April 30 2020 (launch) through 21 February 2021. Primary analyses use raw data, and sensitivity analysis applied survey weights.

#### UMD-CTIS symptomatic never tested but desired testing survey subcohort

On December 21, 2020, additional survey questions were asked of the “never tested” UMD-CTIS respondents regarding whether they had wanted to test in the prior two weeks, and reasons for not getting a test when they wanted one. For the analysis of knowledge-based factors contributing to not getting a test, cross-sectional surveys were limited to a subcohort of those surveys completed from December 21, 2020 onwards (N = 205,017, survey versions 7–9), reporting test-qualifying symptoms in the past 24 hours (N = 32,711, 16.0%), reported having never been tested for coronavirus (N = 12,821, 39.2%) and reported having wanted testing in the prior 14 days (N = 1,956, 15.3%). To describe the factors associated with knowledge barriers to successful testing, we focused on the question “Do any of the following reasons describe why you haven’t been tested for coronavirus (COVID-19) in the last X days?”, where X is symptom duration up to 14 days, and the response option “I don’t know where to go”.

### Data analysis

We calculated the proportions of outcomes among subgroups, considering several outcomes and subgroup definitions. Logistic regression was used to estimate the covariate-outcome association. Covariates considered varied for each analysis as not all variables available in each data set: sex, age, symptom (see Table D in S1 Text), symptom number and duration, symptom-to-survey time, self-reported years of education, index of multiple deprivation [IMD] [15], profession/work, self-reported or national rural-urban classification [RUC] [16]. For some analyses, Zoe reports of either loss of taste/smell or altered taste/smell were combined [16]. Zoe and UMD-CTIS analyses were conducted using Python 3.8 and R 3.6.3, respectively.

### Ethics statement

Boston Children’s Hospital Institutional Review Board (IRB, P00023700) to use UMD Global CTIS data. The UMD IRB (1587016–10) approved the UMD Global CTIS study. King’s College London ethics committee (REMAS ID 18210; LRS-19/20-18210) to use Zoe data. Informed consent for participation in Zoe was provided by the users through the app upon sign-up, while UMD Global CTIS survey was provided by respondents prior to beginning the survey. Informed consent was documented in the digital platform by the respondent (no witness required). This study did not include minors.

## Results

### Quantifying the testing gap

**Fig 1:**
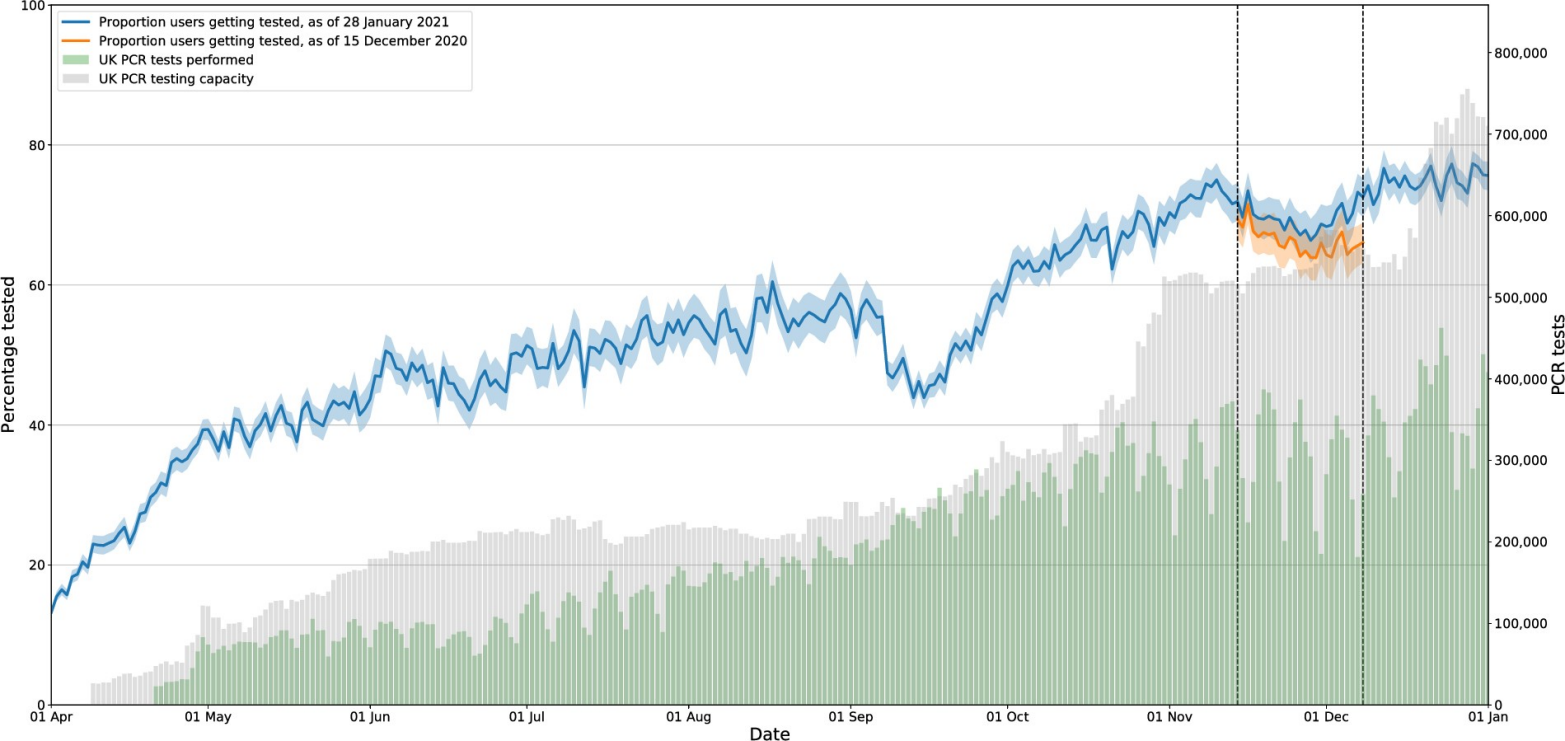
Proportion of participants reporting a COVID-19 test among those reporting test-qualifying symptoms. Proportion of participants reporting at least one test-qualifying symptom for the first time that logged a COVID-19 swab test -7 to +14 days after the onset of the symptom. Dashed vertical lines indicate the three-week study period (14 November 2020 to 8 December 2020) that was used to define participants eligible for a follow-up survey, i.e. those that reported test-qualifying symptoms for the first time but no swab test were invited to the Zoe Follow-Up Survey. Blue line calculated using data logged by 28 January 2020, red line calculated using data logged by 15 December 2020, which was the dataset used to identify target participants for the survey. The difference shows that some participants recorded test data after selection into the follow-up survey cohort. Grey bars indicate the UK PCR testing capacity, while green bars indicate PCR tests performed.

The proportion of Zoe participants reporting COVID-19 testing has increased over time (Fig 1), from <20% (1 April 2020) to >70% (1 January 2021). In mid-September, national PCR testing capacity was exceeded [17], coincident with a transient drop in reported tests. In late 2020, despite adequate test capacity, >25% of test-qualifying app users did not report a test. The UMD-CTIS survey time-series mirrors these trends (Fig A in S1 Text), albeit with a lower absolute proportion, likely due, in part, to survey design differences (e.g. ever tests and symptoms in prior 24 hours, vs tests -7 to +14 days from incident symptoms). The UMD-CTIS testing gap is generally lower amongst respondents with a smartphone, and even lower among symptom-tracking app participants (not necessarily Zoe).

### Symptom severity and not testing

To better understand the factors contributing to COVID-19 testing, Zoe participants with test-qualifying symptoms during the study period, who did not report testing (N = 20,425), were studied further. During this period, the proportion not tested among those with test-qualifying symptoms was higher for those with 1 vs ≥2 test-qualifying symptoms (27.1% vs 14.6%, p<0.001), and for those with symptoms lasting ≤2 days vs >2 (30.1% vs 14.6% p<0.001), (Table 1). Similarly, the proportion of ever-tested in UMD-CTIS was lowest among those with only one test-qualifying symptom or short symptom duration (Fig A in S1 Text). Females with qualifying symptoms were more likely to report not testing than males (26.2% vs 19.7%, p<0.001). Individuals with test-qualifying symptoms and age < 75 were more likely to report not testing than those age ≥ 75 (24.3% vs 16.9% P<0.001) (Table 1). A total of 1,254 users (26.6%) responded to the follow-up survey. Zoe and follow-up survey participants during this period were younger and more female than the general population, similar to the demographic trends reported previously in Zoe [13] and other digital health studies (Table E in S1 Text) [18, 19].

**Table 1:** Characteristics of Zoe CSS app users reporting new-onset test-qualifying symptoms between 14 November and 8 December 2020, and eligible for the Zoe follow-up survey.

		Zoe CSS Data				
		Reported test-qualifying symptoms (14 November—8 December 2020)	Reported and tested		Reported and not tested	
		N	N	%	N	%
**Users**		20,425	15,489/20,425	75.8%	4,936/20,425	24.2%
**Daily reports**		231,678	184,795/231,678	79.8%	46,883/231,678	20.2%
**Age in years**		47.4 (14.1)	47.6 (14.2)		46.8 (13.8)	
**mean (std)**						
	18–24	896	678/896	75.7%	218/896	24.3%
	25–34	2,540	1,866/2,540	73.5%	674/2,540	26.5%
	35–44	4,055	3,067/4,055	75.6%	988/4,055	24.4%
	45–54	4,833	3,674/4,833	76.0%	1,159/4,833	24.0%
	55–64	3,788	2,900/3,788	76.6%	888/3,788	23.4%
	65–74	1,724	1,308/1,724	75.9%	416/1,724	24.1%
	75+	496	412/496	83.1%	84/496	16.9%
	Invalid	25	19/20,425	0.1%	6/20,425	0.0%
**Sex**	Female	13,811	10,186/13,811	73.8%	3,625/13,811	26.2%
	Male	6,569	5,275/6,569	80.3%	1,294/6,569	19.7%
	Other (intersex/prefer not to say)	23	12/23	52.2%	11/23	47.8%
**Test-qualifying symptoms experienced**	C +F + S	960	877/960	91.4%	83/960	8.6%
	C + S	1563	1286/1563	82.3%	277/1563	17.7%
	C + F	881	763/881	86.6%	118/881	13.4%
	F + S	1149	960/1149	83.6%	189/1149	16.4%
	S	5663	4030/5663	71.2%	1633/5663	28.8%
	C	5459	4132/5459	75.7%	1327/5459	24.3%
	F	4577	3314/4577	72.4%	1263/4577	27.6%
**Symptom duration (days)**	7+	1156	1023/1156	88.5%	133/1156	11.5%
	3–5	2806	2445/2806	87.1%	361/2806	12.9%
	2	3490	2878/3490	82.5%	612/3490	17.5%
	1	12619	8823/12619	69.9%	3796/12619	30.1%

Symptom key: F = fever, C = persistent cough, S = loss or altered sense of taste or smell.

### Journey to successful COVID-19 testing

**Fig 2:**
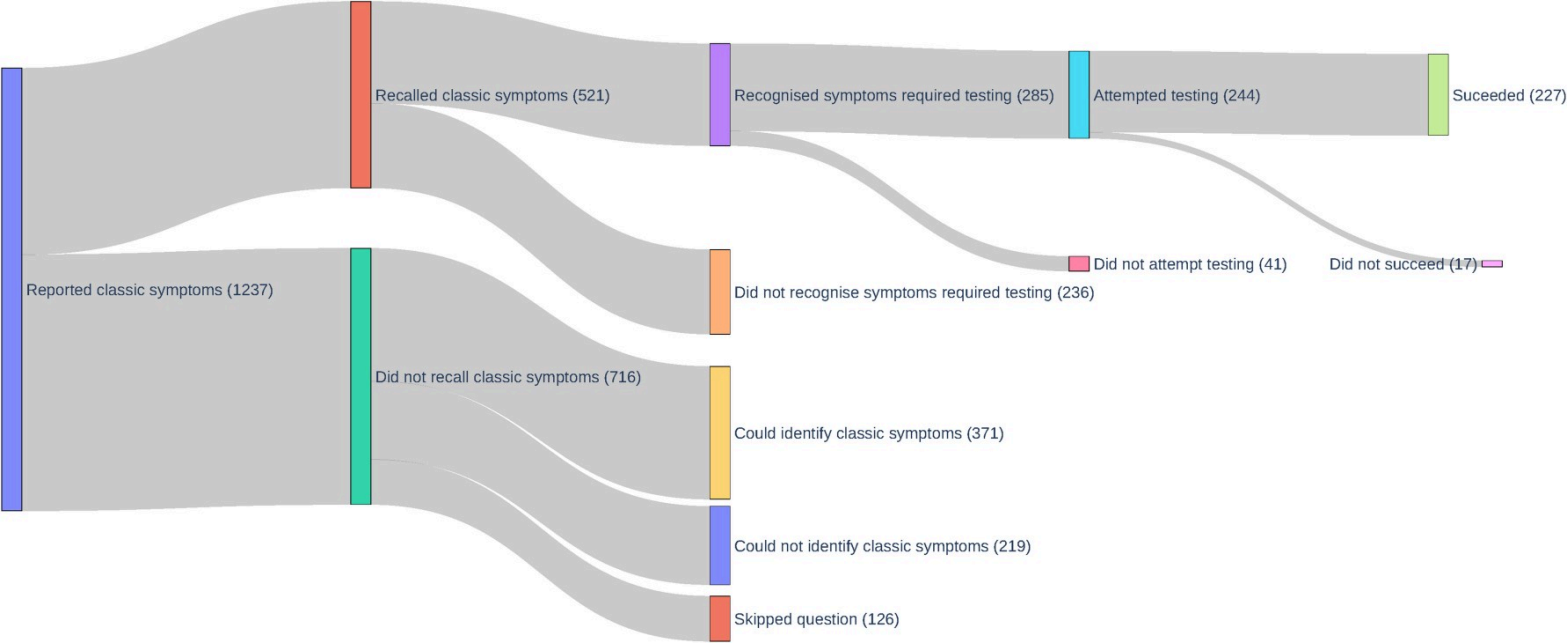
Sankey flow diagram of Zoe follow-up survey responses by stage of the testing journey.

In the Zoe follow-up survey, only 42.1% survey respondents recalled having experienced at least one test-qualifying symptom in the past month (Fig 2). Of those who recalled their symptoms, 54.7% recognised that these symptoms qualified them for a COVID-19 test. Among participants who recognized test-qualifying symptoms, they were likely to go on to attempt (85.6%) and then successfully obtain a COVID-19 test (93.0%, or 18.4% of all survey respondents).

### Recall of previously-reported test-qualifying symptoms

Among those who reported a single test-qualifying symptom (Table 2), recall of absent or altered taste/smell (45.8%) and cough (40.8%), was higher than fever (25.5%), the last of which, as queried in-app (“Do you have a fever or feel too hot?”) was an admixture of two symptoms that may not be recalled equally well. Recall of having experienced test-qualifying symptoms was associated with number of symptoms experienced (per test-qualifying symptom OR = 1.302 [95% CI 1.220–1.391]), symptom duration (per day OR = 1.065 [95% CI 1.054–1.076]) and recency (per symptom-to-survey days OR = 0.995 [95% CI 0.991–1.000]). Number of symptoms and symptom duration remained significantly associated with recall after adjusting for age, sex, and recency of symptom onset (model results in Table F in S1 Text).

**Table 2:** Recall of test-qualifying symptoms in the Zoe follow-up survey.

		Recalled having experienced a test-qualifying symptom
**All users surveyed**		529/1254 (42.2%)
**By symptoms experienced**	C + F + S	22/27 (81.5%)
	C + S	56/66 (84.8%)
	C + F	12/20 (60.0%)
	F + S	19/36 (52.8%)
	S	187/408 (45.8%)
	C	138/338 (40.8%)
	F	83/325 (25.5%)
**By symptom duration**	7+ days	64/69 (92.8%)
	3–5	84/111 (75.7%)
	2	91/184 (49.5%)
	1	274/863 (31.7%)
**By time from symptoms to survey response**	7–14 days	91/176 (51.7%)
	14–21 days	160/383 (41.8%)
	21–28	139/359 (38.7%)
	28 +	131/319 (41.1%)

Symptom key: F = fever, C = persistent cough, S = loss or altered sense of taste or smell.

### Recognizing COVID-19 test-qualifying symptoms

Just 54.7% of those who recalled experiencing test-qualifying symptoms indicated their symptoms qualified them for a COVID-19 test. We queried the remaining respondents (N = 809, i.e. those who did not recall or who did not indicate the symptoms they recalled qualified them for testing) which symptoms would qualify them for a test. These respondents were similarly able to recognize fever (63.4%), cough (67.6%), and loss of smell (65.4%), and much less so for altered smell (29.7%). Only 59.6% identified the triad of fever, cough, and loss of smell as qualifying for a test, with lower recognition amongst the oldest age groups (Table 3). In univariate analyses, each decade older reduced the odds of recognizing the triad (OR = 0.908 [95% CI 0.883–0.933]). This finding remained largely unchanged after adjustment for sex, IMD, and rural-urban living. We found similar associations with the outcome of identifying each individual symptoms (model results in Table G in S1 Text). No associations were found for sex, age, IMD and rural-/urban living.

**Table 3:** Understanding of symptoms qualifying for COVID-19 testing among respondents who did not recall having experienced a test-qualifying symptom.

Group			Recognises symptom as testing-qualifying			
		All of: fever, persistent cough, and loss of smell	Fever	Persistent cough	Loss of smell	Altered smell
**All**		482/809 (59.6%)	513/809 (63.4%)	548/809 (67.7%)	529/809 (65.4%)	240/809 (29.7%)
**Age**	18–24	3/4 (75.0%)	3/4 (75.0%)	4/4 (100.0%)	4/4 (100.0%)	3/4 (75.0%)
	25–34	39/49 (79.6%)	40/49 (81.6%)	41/49 (83.7%)	40/49 (81.6%)	21/49 (42.9%)
	35–44	99/135 (73.3%)	102/135 (75.6%)	113/135 (83.7%)	109/135 (80.7%)	38/135 (28.1%)
	45–54	143/217 (65.9%)	152/217 (70.0%)	160/217 (73.7%)	156/217 (71.9%)	66/217 (30.4%)
	55–64	118/223 (52.9%)	129/223 (57.8%)	137/223 (61.4%)	132/223 (59.2%)	67/223 (30.0%)
	65–74	72/150 (48.0%)	79/150 (52.7%)	82/150 (54.7%)	78/150 (52.0%)	39/150 (26.0%)
	75+	8/31 (25.8%)	8/31 (25.8%)	11/31 (35.5%)	10/31 (32.3%)	6/31 (19.4%)

### Reasons for not testing among those who qualified for and wanted a test

In the follow-up survey, the proportion of respondents who recognised their symptoms qualified them for testing, and attempted, but did not succeed at testing was low (7%, N = 17 of 244, Fig 2). Due to limited sample size of this path to testing, other barriers could not be evaluated. We therefore evaluated complementary data from a subcohort of UMD-CTIS respondents from 21 December 2020 to 21 February 2021, who endorsed test-qualifying symptoms, who had never tested, and who indicated “yes” to the question “Have you wanted to get tested for coronavirus (COVID-19) at any time in the last 14 days?” (N = 1,956, Table 4). Among those who wanted testing, “I don’t know where to go” was the most frequently selected option (32.4%). The other multi-choice reasons were: “I am unable to travel to a testing location” (29.1%), “I tried to get a test but was not able to get one” (25.6%), “I am worried about bad things happening to me or my family (including discrimination, government policies, and social stigma)” (18.4%), “I can’t afford the cost of the test” (17.9%), and “I don’t have time to get tested”(13.3%). Given the scope of this study, we have focused on the knowledge-based response, though we acknowledge the logistical barriers are important.

**Table 4:** Characteristics of UK Global COVID-19 Trends and Impacts Survey (UMD-CTIS) survey respondents reporting test-qualifying symptoms, never testing, but wanting to test in the prior 14 days, for surveys between 21 December 2020 through February 21, 2021.

		UK Global COVID-19 Trends and Impacts Survey Subcohort: Reported test-qualifying symptoms, never tested, but test wanted			
		All	Did not know where to test (Missing)	Did not know where to test (No)	Did not know where to test (Yes)
		Number (Percent, %)	Number (Percent, %)	Number (Percent, %)	Number (Percent, %)
**Cross-Sectional Surveys**		1,956	479/1956 (24.5%)	844/1956 (43.1%)	633/1956 (32.4%)
Age Group*	18–24	207/1,956 (10.6%)	27/207 (13.0%)	107/207 (51.7%)	73/207 (35.2%)
	25–34	321/1,956 (16.4%)	43/321 (13.4%)	184/321 (57.3%)	94/321 (29.3%)
	35–44	271/1,956 (13.9%)	54/271 (19.9%)	141/271 (52.0%)	76/271 (28.0%)
	45–54	321/1,956 (16.4%)	95/321 (29.6%)	130/321 (40.4%)	96/321 (29.9%)
	55–64	303/1,956 (15.5%)	80/303 (26.4%)	101/303 (33.3%)	122/303 (40.3%)
	65–74	159/1,956 (8.13%)	45/159 (28.3%)	51/159 (32.1%)	63/159 (39.6%)
	75+	55/1,956 (2.81%)	15/55 (27.2%)	14/55 (25.5%)	26/55 (47.3%)
	Not answered	319/1,956 (16.3%)	120/319 (37.5%)	116/319 (36.4%)	83/319 (26.0%)
Sex*	Female	836/1,956 (42.7%)	204/836 (24.4%)	383/836 (45.8%)	249/836 (29.8%)
	Male	733/1,956 (37.5%)	141/733 (19.2%)	317/733 (43.2%)	275/733 (37.5%)
	Other	21/1,956 (1.07%)	2/21 (9.5%)	10/21 (47.6%)	9/21 (42.9%)
	Prefer not to say	17/1,956 (0.869%)	4/17 (23.5%)	5/17 (29.4%)	8/17 (47.1%)
	Not answered	349/1,956 (17.8%)	128/349 (36.7%)	129/349 (37.0%)	92/349 (26.4%)

* Survey-weighted mean age 39.3 years (unweighted 45.4 years) and proportion female of all male and female respondents 51.9%.

#### Not knowing where to test among the symptomatic wanting testing

**Fig 3:**
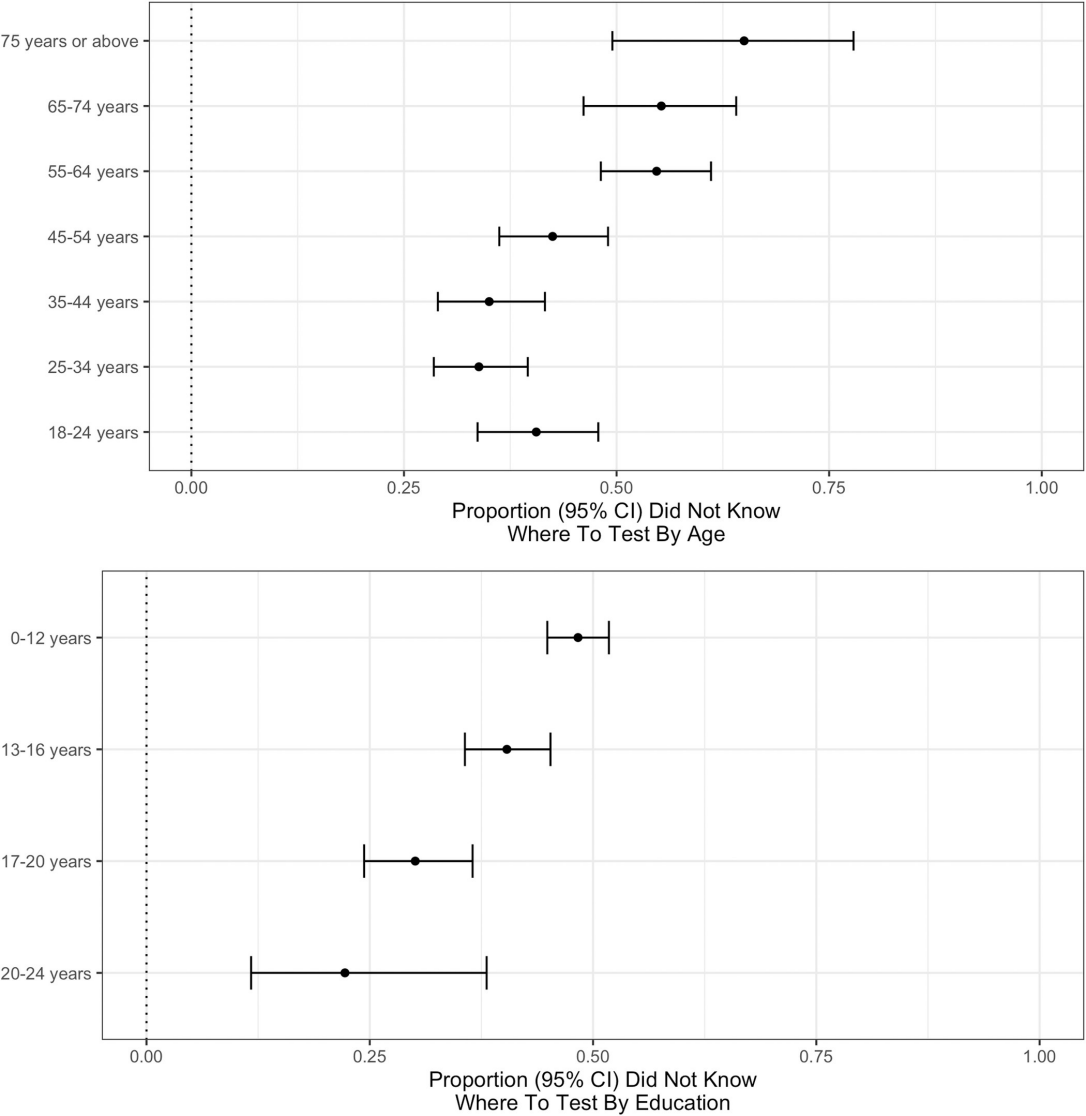
Not knowing where to get a COVID-19 test stratified by age and education. Proportion (95% confidence interval) of respondents who indicated “yes” (per “yes” plus “no”) for “I don’t know where to go” as an option to the question “Do any of the following reasons describe why you haven’t been tested for coronavirus (COVID-19) in the last X days?”, where X is the self-reported duration of symptom capped at 14 days. This was restricted to untested respondents with test-qualifying symptoms. Proportions stratified by age (top) and education (bottom).

We further investigated demographic factors associated with not knowing where to go to obtain a test (Fig 3). Not knowing where to go to obtain a test (“yes” vs referent “no”) was associated with older age (per decade OR = 1.207 [1.129–1.292]) and less education (per 4-years OR = 0.685 [0.599–0.783]). Male sex (OR = 1.334 [1.064–1.675]) and living outside a city (OR = 1.201 [0.926–1.562]) were not significant with Bonferroni correction for multiple hypothesis testing (p-threshold 0.0125 = 0.05/4). Education was similarly protective for not knowing where to test adjusting for age and sex, use of survey weights, or assuming missing responses were “no” (models results in Table H in S1 Text).

Acknowledging our limited sample size, we conducted qualitative, hypothesis-generating analyses of other demographic factors correlated with knowledge barriers (Fig B in S1 Text). While cities were not protective in the regression model, the proportion not knowing where to test was slightly lower in London than elsewhere (Fig C in S1 Text). Having a smartphone and using a symptom-tracking app qualitatively had a bigger impact on the knowledge gap than the relatively small urban-rural and regional differences. There were modest qualitative differences in not knowing where to test by profession, with the highest proportions among those in transportation, tourism and construction and the lowest in finance, public administration and health.

## Discussion

### Persistent testing gap

Our analysis finds that in December 2020 approximately one quarter of symptomatic UK Zoe participants who qualified for a COVID-19 test did not undergo testing. The proportion of ever-tested recently-symptomatic UK UMD-CTIS respondents echoes this trend. While we show a substantial improvement from April, 2020, the persistent testing gap is problematic for pandemic management in the UK and elsewhere. Non-pharmaceutical mitigation strategies are likely to be required [20–22], despite effective vaccines, because of COVID-19 transmissibility and the anticipated time to reach herd immunity, even with a one-dose immunization strategy [23]. The lower the proportion of identified infections, such as through insufficient testing of symptomatic cases, the more likely transmission events will go unchecked.

### Knowledge barriers to testing

With this testing gap in mind, we sought to characterize barriers to testing that might inform improvements to the UK testing programme using data from two large, complementary surveillance platforms. Through analysis of prospective self-reported testing outcomes, along with follow-up and population-sampled surveys, we identified three key knowledge barriers along the path to successful COVID-19 testing.

Firstly, the association of less testing with brief and/or single test-qualifying symptoms suggests an implied severity threshold for testing that is inconsistent with guidance [5], and with the spectrum of infectious COVID-19; given those with asymptomatic disease can transmit, it is reasonable to expect that those experiencing just a single symptom, or brief duration of symptoms, are able to transmit and need to be tested [24]. Individuals may minimise their symptoms, particularly if they perceive their risk of contracting COVID-19 as low, or possibly hold the misconceived notion that COVID-19 manifests in a stereotypical manner [25]. In a preprint DHSC report utilizing online surveys May 25 to August 5, 2020, those with test-qualifying symptoms did not request testing because symptoms were mild (16.0%), improved (16.1%), or they did not think symptoms were due to COVID-19 (20%) [8]. Secondly, we show that four of ten did not recognize all three of the triad of UK test-qualifying—i.e. fever, cough and loss of smell. The DHSC report [8] estimated 51.1% of all respondents failed to recognize the triad. Despite increased press coverage, across newspapers, TV, and radio, and a second wave with mitigation intensification [26], recognition of these test-qualifying symptoms in our survey around six months later increased by ~10% and therefore remains alarmingly low. Lastly, one third of those who wanted a COVID-19 test cited not knowing where to test as a contributing factor to their not getting one. This was the most frequently cited reason among the six options. Thus, despite the fact that survey respondents are possibly more aware of health information, they acknowledge challenges to finding testing that are comparable to, if not greater than, logistical barriers to testing (e.g. travel, time), even within the framework of the more centralized UK testing programme.

### Public health implications

Our findings have significant public health implications. The UK NHS testing programme offers free COVID-19 tests to those with test-qualifying symptoms, with the list of qualifying symptoms unchanged since loss/alteration to taste and smell were included on 18 May 2020 [27], and tests accessed through a central booking system [5]. Risk mitigation and public health principles generally would agree with these key features of the UK program i.e. the use of concise and consistent guidance, and limiting logistical barriers to following guidance. In this sense, the UK is a sort of case study of the “best case scenario”, and yet there is still a significant gap in understanding. Other testing barriers do exist and should be addressed. Nevertheless, we highlight through this study that, for many with test-qualifying symptoms, knowing when to test is an early essential factor and knowing where to test is a later contributing factor to inadequate testing of potential COVID-19 cases. Not only are greater efforts needed to educate the UK public, it is likely that comparable efforts to mind the knowledge gap will be needed in countries with regionally varying testing criteria or methods of accessing testing.

Our work suggests there is a need for messaging improvements to the UK testing campaign. In our study, among the untested who qualified for a test, older age was associated with not knowing when and where to test. In the earlier DHSC report [8], older age was generally protective with respect to testing knowledge and behaviors, perhaps suggesting knowledge gains in the young over the past six months. Fewer years of education was also associated with not knowing where to test in our study. We found suggestive evidence that the knowledge gap may be more pronounced among those who do not have smartphones. Older populations in pre-pandemic studies have slower adoption of certain technologies, yet the abrupt social isolation resulting from mitigation strategies may be leaving important segments of the population behind [28]. Education attained and age are likely not the root cause of not knowing when and where to test. Rather they likely highlight pre-existing health-information disparities that have been exacerbated by a year of unprecedented changes in how individuals interface with each other and the world.

Our findings support the need for targeted messaging to certain at-risk demographic groups, possibly in a non-digital format (e.g. radio, community signage). These efforts would be aided by further research seeking to understand where people obtain their information about COVID-19—information we did not collect in our study. Our findings are particularly timely in light of work showing that expansion of the symptoms that qualify for a test would help detect more cases, assuming those who qualify do indeed successfully test [29, 30]. This theoretical gain in case detection could be lost if the change in tack leaves vulnerable populations behind. There is overlap between knowledge risk factors and COVID-19 risk, such as older age [31], though we did see a higher absolute rate of testing in the oldest age group. Overlap with vaccine hesitancy risk factors may further amplify disparities in healthcare access, leaving some groups both less tested and less protected.

Furthermore, messaging could also emphasise that even individuals with mild or transient symptoms may have COVID-19 and should get tested. COVID-19 has a broad spectrum of disease severity with a substantial number of cases being fully asymptomatic, and with asymptomatic carriers still being able to transmit, albeit at reduced rates [24].

### Strengths and limitations

The Zoe platform affords a unique opportunity to prospectively link testing behaviours with incident symptoms in a large user base comprising ~6% of the UK population. The UMD-CTIS platform, though smaller in size and slightly different in survey design, corroborates temporal trends over in the broader population. To our knowledge, this has enabled the first time-varying estimate of testing rates amongst individuals that qualify for COVID-19 tests over the course of the pandemic. Both platforms could be leveraged to track the testing and knowledge gaps, in real time, allowing the effectiveness of interventions, such as improved messaging on when and where to test, to be assessed.

We acknowledge a number of limitations to this study. Digital surveys include selected populations not necessarily representative of the wider population. Such platforms have well-documented biases in demographic age, sex, and socioeconomic factors which we adjusted for in our analyses [18, 32]. In addition, digital surveys may not be generalizable, as they may be enriched for health-conscious internet-connected participants, and thus underestimate disparities in at-risk demographic groups. We show that symptom-tracking app participants and those with smartphones have higher testing rates than all UMD-CTIS survey respondents.

Confounding and measurement bias in this observational study using self-reported covariates and outcomes may also cause us to miss other important issues related to testing. We adjusted for common confounders, and attempted to identify proxies for the knowledge gap rather than attribute causality. There is no timely, efficient trial to conduct analyses of this scale. Self-report could introduce non-differential and differential measurement error, including the possibility of some events being omitted, or recorded inaccurately or inappropriately. Furthermore, the financial implications of having to self-isolate disproportionately affect the poorest, and may increase unwillingness to test [33] and respondents may be wary of self-reporting socially stigmatized reasons for not complying with guidance. We examined the association between knowledge barriers and level of education and profession, but were not able to examine other socioeconomic factors, such as income. Furthermore, this study does not explore the joint effect of multiple barriers (knowledge, logistical, financial or other), nor the potential benefit of facilitators. Lastly, selection can theoretically induce collider bias [34] if the exposure and outcome are both causes of participation or subpopulation selection.

## Conclusion

Testing is a fundamental principle of population-wide transmission mitigation. While the UK now has sufficient testing capacity, consistent guidelines, and free testing for those who qualify that is coordinated centrally, still we see a 25% testing gap among those with test-qualifying symptoms. We show this gap may be driven in part by a lack of understanding of mild COVID-19, national testing criteria, and testing access, especially among the elderly and those who have had fewer years of education. We propose altering the course of the UK testing programme to address this knowledge barrier to COVID-19 testing. In addition, other countries may benefit from improved understanding of modifiable barriers.

## Supporting information

S1 Text Supplementary text including the following supplementary tables and figures. **Table A: Zoe CSS questions. Table B:** Zoe CSS testing survey questions. **Table C:** UMD Global COVID-19 Trends and Impact Survey questions. **Table D:** Comparison of question wording between Zoe and UMD-Facebook. **Figure A:** Testing trends. **Table E:** Comparison of Zoe survey respondents to all surveyed. **Table F:** Ability to recall symptoms in Zoe CSS survey. **Table G:** Understanding of testing criteria. **Table H:** Knowing where to test. **Figure B:** Qualitative demographic-knowledge relationships in UMD-CTIS. **Figure C:** Participant locations for UMD-CTIS.
(PDF)
