# Supplementary material for: Knowledge barriers in a national symptomatic-COVID-19 testing programme
Source: PLOS Glob Public Health. 2022 Jan 19;2(1):e0000028. doi: 10.1371/journal.pgph.0000028 (PMC10022193; doi:10.1371/journal.pgph.0000028)
Supplement: S1 Text — Table A: Zoe CSS questions. Table B: Zoe CSS testing survey questions. Table C: UMD Global COVID-19 Trends and Impact Survey questions. Table D: Comparison of question wording between Zoe and UMD-Facebook. Figure A: Testing trends. Table E: Comparison of Zoe survey respondents to all surveyed. Table F: Ability to recall symptoms in Zoe CSS survey. Table G: Understanding of testing criteria. Table H: Knowing where to test. Figure B: Qualitative demographic-knowledge relationships in UMD-CTIS. Figure C: Participant locations for UMD-CTIS. (PDF) [file pgph.0000028.s001.pdf]

Supplementary Text

Table A: Zoe CSS questions

In each daily report, users are asked about any symptoms they experience that day, and any COVID-19 tests they have had.

Symptoms
Users are asked “How do you feel physically right now?”. If they select “I’m not feeling quite right”, they are presented with a symptom options checklist. The symptom options specifically analysed in this study are:

Are you experiencing any of the below symptoms?

- ❏ Fever or feel too hot
- ❏ Loss of smell / taste
- ❏ Altered smell / taste (things smell or taste different to usual)
- ❏ Persistent cough (coughing a lot for more than an hour, or 3 or more coughing episodes in 24 hours)

Testing
Users are shown a list of all COVID-19 tests they have logged through the app. They are able to add new tests, edit existing entries, or select “This list is correct”. If a user chooses to add a new test, they are asked:

- Do you know the date of your test?
  - If yes, select date
- How was the test performed?
  - A swab of my nose or throat
  - I spat in a cup/tube
  - A finger-prick blood test
  - A blood test, done using a needle
  - Other, please specify (free text)
- Where was this test performed?
  - At Home
  - Drive-through Regional Testing Centre
  - Hospital (not drive-through)
  - GP
  - Chemist / Pharmacy
  - Work (excluding hospital or GP)
  - Other, please specify
- What are the results of this test?
  - Negative
  - Positive

- Not clear/ failed
- Waiting for results

Table B: Zoe CSS testing survey questions

Q1 Have you experienced any of the following symptoms in the last month? (check all that apply)

- ❏ Fever
- ❏ Persistent cough
- ❏ Loss of smell or taste
- ❏ Altered smell or taste
- ❏ Shortness of breath
- ❏ Fatigue
- ❏ Muscle or body aches
- ❏ Headache
- ❏ Sore throat
- ❏ Congestion or runny nose
- ❏ Nausea or vomiting
- ❏ Diarrhea
- ❏ None of the above

If None of the above; Proceed to **Q2a** then **SURVEY END**.
Else; Proceed to **Q2.**

Q2 Did these symptoms qualify you for a COVID-19 swab test where you live?

- ❏ Yes
- ❏ No
- ❏ Do not know

If YES; Proceed to **Q3**
If NO or DO NOT KNOW; Proceed to **Q2a**

Q2a What symptoms qualify you for a covid test where you live? (check all that apply)

- ❏ Fever
- ❏ Persistent cough
- ❏ Loss of smell or taste
- ❏ Altered smell or taste
- ❏ Shortness of breath

- ❏ Fatigue
- ❏ Muscle or body aches
- ❏ Headache
- ❏ Sore throat
- ❏ Congestion or runny nose
- ❏ Nausea or vomiting
- ❏ Diarrhea
- ❏ None of the above
- ❏ Do not know

Proceed to **Q3** (if arrived here directly from Q1, proceed to **SURVEY END**)

Q3 Did you try to get a swab test?

If YES; Proceed to **Q4**

If NO; Proceed to **Q3A**

Q3a Please select the reason(s) you did not attempt to get a swab test (all that apply)

- ❏ I did not believe I could/should get a test with my symptoms at the time
- ❏ My symptoms were normal / not new for me
- ❏ I thought that travelling to a testing appointment would be difficult or risky
- ❏ I was concerned about discomfort or pain from the swab
- ❏ I have already had Covid and did not think I could get it again
- ❏ I couldn't find the time to go to an appointment (e.g. couldn't get time off work/childcare)
- ❏ I was concerned testing positive might affect me financially (job/income/studies)
- ❏ I was concerned about the cost of the the test
- ❏ I was concerned testing positive would affect me socially (time away from friends/family)
- ❏ I was concerned about testing positive and being contacted by contact tracers or health authorities
- ❏ The tests are not reliable
- ❏ OTHER - free text

Proceed to **SURVEY END**

Q4 Did you receive a COVID-19 test (swab or otherwise) within 14 days of having such symptoms?

If YES; Proceed to **Q5**

If NO, Proceed to **Q4a**

Q4a TRIED TO GET TEST REASONS

Please state the reason(s) the test did not happen (all that apply):

- ❑ I did not know how to get a test
- ❑ There were no testing appointments available
- ❑ There were no home testing kits available
- ❑ The test never arrived/I never received the result back
- ❑ I thought that travelling to a testing appointment would be too risky
- ❑ I was concerned about swabbing myself/being swabbed
- ❑ Difficult with transportation to my appointment (e.g. too far away/I didn't have a vehicle)
- ❑ I couldn't find the time to go to an appointment (e.g. couldn't get time off work/childcare)
- ❑ I could not afford the test
- ❑ I was told by a doctor or testing centre that I didn't need a test
- ❑ OTHER - free text

Proceed to **SURVEY END**

Q5 What date was your COVID-19 test? Please also log this test through the app, if you have not done so already.

[Date entry field]

Proceed to **SURVEY END**

SURVEY END

Table C: UMD Global COVID-19 Trends and Impact Survey questions

Covariate	Survey Question	Model notes on exclusions, variable coding, and survey
Survey Version	Survey wave 7, 8 and 9 (Pre-wave 7 used for time series)	Survey wave 7 launched December 21, 2020, wave 8 launched January 14, 2021, and wave 9 launched February 6, 2021.
Country	What is the country or region where you are currently staying?	ISO country data included: GBR
Region	What is the administrative region where you are currently staying?	Regions included:<br>East Midlands<br>East of England (or East Anglia)<br>London (or Greater London)<br>North East<br>North West<br>Northern Ireland<br>Scotland<br>South East<br>South West<br>Wales<br>West Midlands<br>Yorkshire and the Humber
Symptoms	In the last 24 hours, have you had any of the following? [Respondents may select none or up to 14 symptoms]<br>1 = Yes<br>2 = No<br>-99 = missing/valid skipped/invalid<br>-77 = seen but unanswered	Symptoms included:<br>Fever<br>Cough<br>Loss of smell or taste
Duration of symptoms	For how many days have you had at least one of these symptoms?<br>[Ask if selected choices count greater than or equal to 1]	[OPEN RESPONSE: NUMBER VALIDATION]
Testing	Have you ever been tested for coronavirus (COVID-19)?<br>1 = Yes<br>2 = No<br>-99 = missing/valid	

	skipped/invalid<br>-77 = seen but unanswered	
Want testing	Have you wanted to get tested for coronavirus (COVID-19) at any time in the last 14 days [asked if No is selected in the Testing question?<br>1 = Yes<br>2 = No<br>-99 = missing/valid skipped/invalid<br>-77 = seen but unanswered	
Reason(s) for not testing	Do any of the following reasons describe why you haven't been tested for coronavirus (COVID-19) in the last [feed days back - cap at 14] days? [y/n for any of 6 options, asked if Want testing question answer Yes]<br>1 = Yes<br>2 = No<br>-99 = missing/valid skipped/invalid<br>-77 = seen but unanswered<br><br>[“feed days back” from Duration of Symptom question]	Options (respondents may select more than one):<br>❏ I tried to get a test but was not able to get one.<br>❏ I don't know where to go.<br>❏ I can't afford the cost of the test.<br>❏ I don't have time to get tested.<br>❏ I am unable to travel to a testing location (including because of transportation cost, safety, or physical limitations).<br>❏ I am worried about bad things happening to me or my family (including discrimination, government policies, and social stigma).
Sex	What is your gender?<br>Male<br>Female<br>Other<br>Prefer not to answer<br>missing/valid skipped/invalid<br>seen but unanswered	Modeled as female referent vs male (other categories excluded when sex in the model)
Age	What is your age?<br>18-24 years<br>25-34 years<br>35-44 years	Modeled as linear by age with 18-24 years referent, assuming age in years:<br>18-24 years as 20 years

	45-54 years<br>55-64 years<br>65-74 years<br>75 years or older<br>missing/valid skipped/invalid<br>seen but unanswered	25-34 years as 30 years<br>35-44 years as 40 years<br>45-54 years as 50 years<br>55-64 years as 60 years<br>65-74 years as 70 years<br>75 years or older as 80 years<br><br>Sensitivity analysis with 18-54 years referent vs >=55 years.
Education	How many years of education have you completed?<br>OPEN RESPONSE: NUMBER VALIDATION<br>-99 = missing/valid skipped/invalid<br>-77 = seen but unanswered	Restricted to 0-24 years in responses.<br><br>Modeled in 4-year categories with <=12 years as referent, 13-16, 17-20, and 21-24 corresponding generally to secondary, post-secondary, and graduate education. These roughly corresponded to quartiles of the distribution.<br><br>Sensitivity analyses using referent of 0-8, 9-16, and 17-24 years.
Area staying	Which of these best describes the area where you are currently staying?<br>City<br>Town<br>Village or rural area<br>missing/valid skipped/invalid<br>seen but unanswered	Modeled at City referent vs Town and Village/Rural area.
Work Type	[if D7 == Yes, In the last 7 days, did you do any work for pay, or do any kind of business, farming, or other activity to earn money, even if only for one hour?]<br><br>What is the main activity of the business or organization in which you work?<br><br>[if D8 == Yes, Before February 2020, were you	Options:<br>Agriculture<br>Buying and selling<br>Construction<br>Education<br>Electricity/water/gas/waste<br>Financial/insurance/real estate services<br>Health<br>Manufacturing<br>Mining<br>Personal services<br>Professional/scientific/technic

	working for pay, or doing any kind of business, farming, or other activity to earn money?] What is the main activity of the business or organization in which you were working before February 2020?	al activities<br>Public administration<br>Tourism<br>Transportation<br>Other<br>missing/valid skipped/invalid<br>seen but unanswered
Weight	*survey weight to adjust from FB user population to the general population*	

Table D: Comparison of question wording between Zoe and UMD-Facebook

Comparison for all symptoms considered as test-qualifying in this work.

**Zoe app**	**Zoe follow-up survey**	**UMD-Facebook**
Fever or feel too hot	Fever	Fever
Persistent cough (coughing a lot for more than an hour, or 3 or more coughing episodes in 24 hours)	Persistent cough	Cough
Loss of smell / taste	Loss of smell or taste	Loss of smell or taste
Altered smell / taste (things smell or taste different to usual)	Altered smell or taste	
Symptoms logged daily.<br>Tests may be logged at any time.<br>Tests considered if -7 to +14 days of first test-qualifying symptom	NA	Cross-sectional survey queried symptoms in prior 24 hours, duration of any symptoms that were selected, ever testing, and wanting to test in the prior X days.<br><br>X = duration of symptoms self-reported up to 14 daily

Figure A: Testing trends

UMD-CTIS temporal trends in the proportion of symptomatic survey respondents ever tested from survey start (April 30, 2020). Additional questions for the never tested respondents were added in survey wave 7 and beyond (December 21, 2020, vertical grey line). Surveys with non-missing geographic region in the UK, self-reporting at least one of fever, cough or loss of smell/taste in the prior 24 hours (N=107,021, solid purple), plotting proportion (Wilson method for binomial 95% confidence intervals) who indicate ever testing for COVID-19. (a) Starting June 27, 2020, respondents were queried about technology use. There were N=67508 (63.1%, dashed green) who reported using a smartphone device, while N=16,488 (15.4%, dashed yellow) reported using any symptom tracking app. Survey-weighted mean (+/- 2 standard deviation) for all surveys with test-qualifying symptoms included for comparison. This was generally lower than the raw proportion ever tested. Testing proportion among cross-sectional surveys varied by (b) number of test-qualifying symptoms and (c) duration of symptoms (for those who reported symptom duration), similar to the prospective, longitudinal Zoe app findings.

(a) Subgroup analysis by technology use.

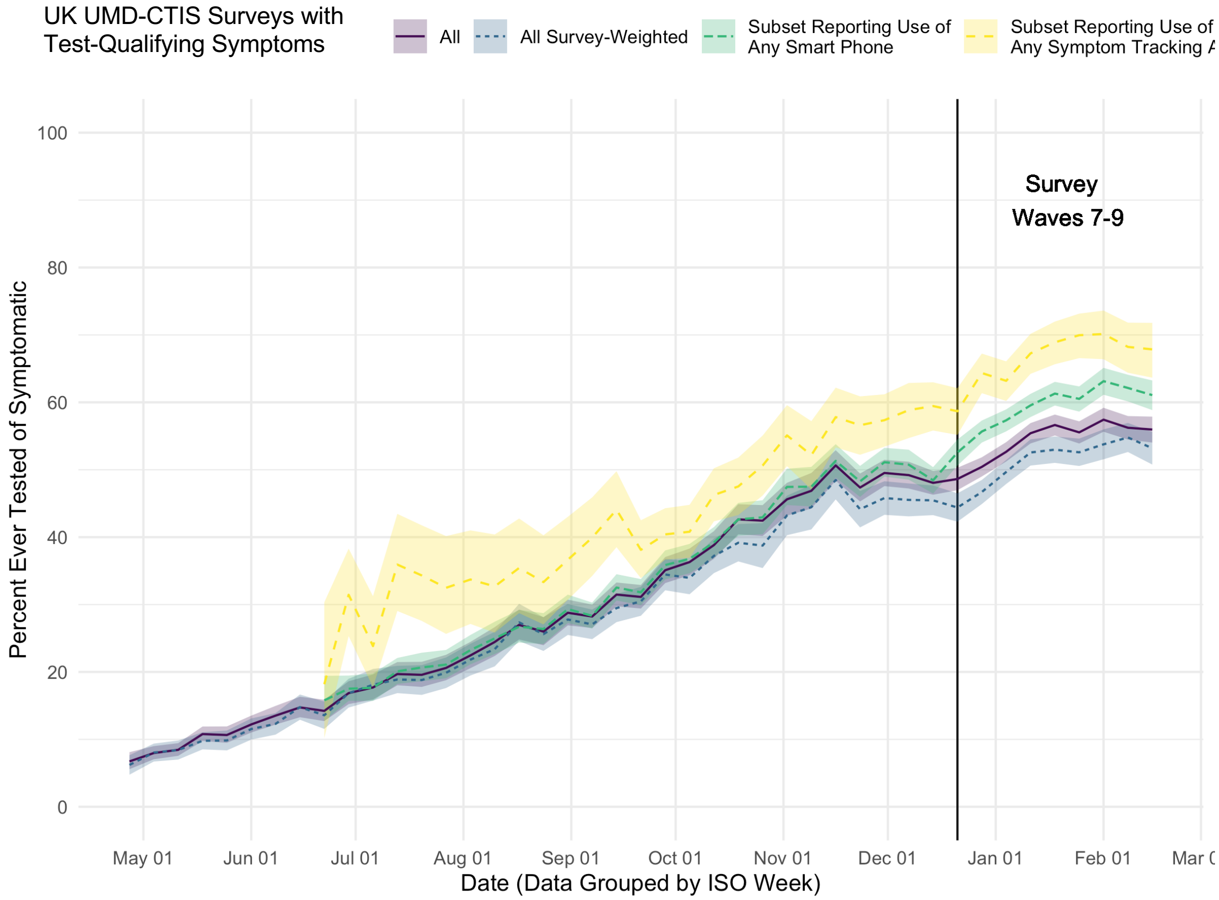

(b) Subgroup analysis by number of test-qualifying symptoms.

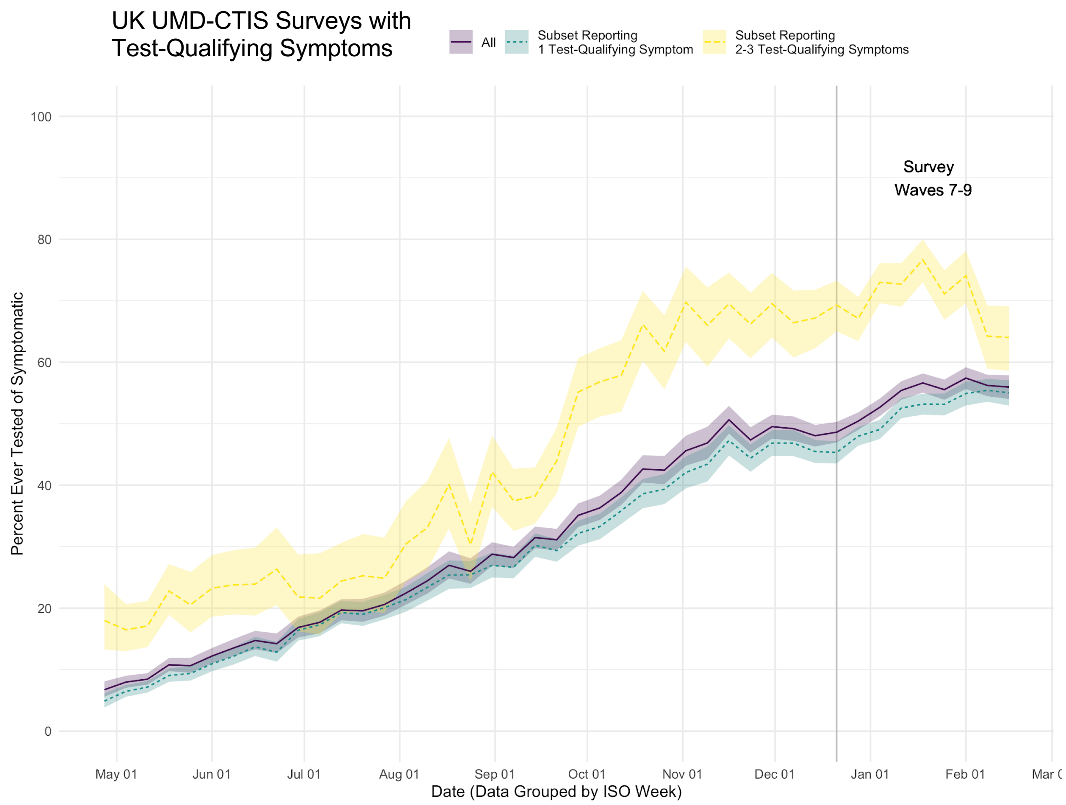
UK UMD-CTIS Surveys with
Test-Qualifying Symptoms
All
Subset Reporting
1 Test-Qualifying Symptom
Subset Reporting
2-3 Test-Qualifying Symptoms
Survey
Waves 7-9
Percent Ever Tested of Symptomatic
100
80
60
40
20
0
May 01
Jun 01
Jul 01
Aug 01
Sep 01
Oct 01
Nov 01
Dec 01
Jan 01
Feb 01
Date (Data Grouped by ISO Week)

(c)

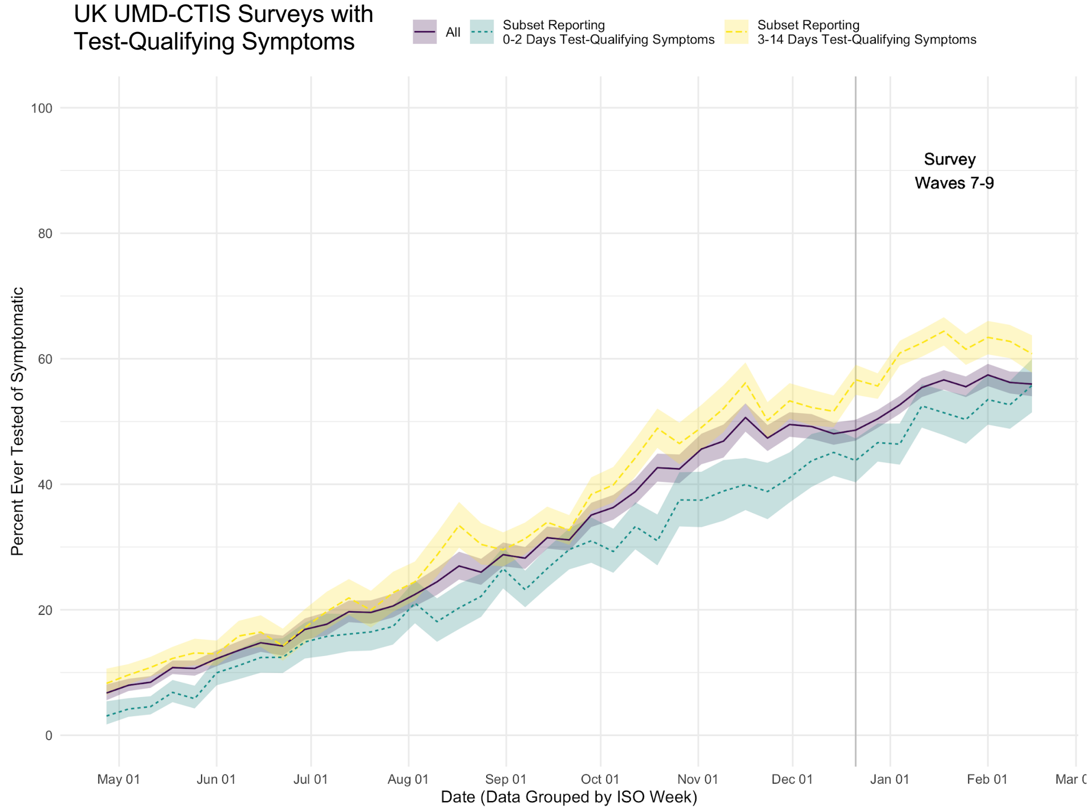
UK UMD-CTIS Surveys with
Test-Qualifying Symptoms
All
Subset Reporting
0-2 Days Test-Qualifying Symptoms
Subset Reporting
3-14 Days Test-Qualifying Symptoms
Survey
Waves 7-9
Percent Ever Tested of Symptomatic
100
80
60
40
20
0
May 01
Jun 01
Jul 01
Aug 01
Sep 01
Oct 01
Nov 01
Dec 01
Jan 01
Feb 01
Date (Data Grouped by ISO Week)

Table E: Comparison of Zoe survey respondents to all surveyed.

Comparison between all users sent the Zoe follow-up survey and respondents.

		Zoe CSS Data			
		**Reported and not tested (sent survey)**		**Responded to survey**	
		**N**	**%**	**N**	**%**
**Users**		4,936		1,239	
**Daily reports**		46,883		17,088	
**Age in years mean (std)**		46.8 (13.8)		53.1 (12.6)	
	18-24	218/4,936	4.4%	9/1,239	0.7%
	25-34	674/4,936	13.7%	67/1,239	5.4%
	35-44	988/4,936	20.0%	195/1,239	15.7%
	45 - 54	1,159/4,936	23.5%	299/1,239	24.1%
	55 - 64	888/4,936	18.0%	314/1,239	25.3%
	65 - 74	416/4,936	8.4%	195/1,239	15.7%
	75+	84/4,936	1.7%	38/1,239	3.1%
	Invalid	6/4,936	0.1%	1/1,239	0.1%
**Sex**	Female	3,625/4,936	73.4%	943/1,239	76.1%
	Male	1,294/4,936	26.2%	295/1,239	23.8%
	Other (intersex/prefer not to say)	11/4,936	0.2%	0	0.0%
**Test-qualifying symptoms experienced**	C + F + S	83/4936	1.7%	27/1237	2.2%

	C + S	277/4936	5.6%	66/1237	5.3%
	C + F	118/4936	2.4%	20/1237	1.6%
	F + S	189/4936	3.8%	36/1237	2.9%
	S	1633/4936	33.1%	408/1237	33.0%
	C	1327/4936	26.9%	338/1237	27.3%
	F	1263/4936	25.6%	325/1237	26.3%
**Symptom duration (days)**	7+	133/4936	2.7%	69/1237	5.6%
	3-5	361/4936	7.3%	111/1237	9.0%
	2	612/4936	12.4%	184/1237	14.9%
	1	3796/4936	76.9%	863/1237	69.8%

Table F: Ability to recall symptoms in Zoe CSS survey.

Logistic regression models for the association of ability to recall test-qualifying symptoms, including covariates of sex, age, symptom duration, number of test-qualifying symptoms, and time between symptom onset and survey response.

		Odds Ratios				
**Model**	**Number**	**Sex**	**Age**	**Symptom duration**	**Number test-qualifying symptoms**	**Time from symptom onset**
Univariate Sex (Female Referent)	1	1.048 (0.980-1.121) p=0.170	---	---	---	--
Univariate Age (Per Decade Above 18-24 Referent)	2	---	1.005 (0.982-1.028) p=0.660	---	---	---
Univariate Symptom duration (Per day above 0)	3	---	---	**1.065 (1.054-1.076) p<0.001**	---	---
Univariate number of test-qualifying symptoms (1 Referent)	4	---	---	---	**1.302 (1.220-1.391) p<0.001**	---
Univariate time from symptom onset to survey (Per day, 10 days referent)	5	---	---	---	---	**0.995 (0.991-1.000) p=0.029**
Multivariate	6	0.947 (0.888-1.009) p=0.091	0.997 (0.975-1.018) p=0.760	**1.062 (1.049-1.074) p<0.001**	**0.991 (0.987-0.994) p<0.001**	**1.142 (1.067-1.223) p=0.000**

Table G: Understanding of testing criteria.

Lgistic regression models for the association of ability to recall test-qualifying symptoms, including covariates of sex, age, index of multiple deprivation, rural-urban classification

			Odds Ratios			
**Symptoms**	**Model**	**Number**	**Sex**	**Age**	**IMD**	**RUC**
All three (fever, cough, loss of smell)	Univariate Sex (Female Referent)	1	0.927 (0.853-1.007) p=0.072	---	---	---
	Univariate Age (Per Decade Above 18-24 Referent)	2	---	**0.908 (0.883-0.933) p=0.000**	---	---
	Univariate IMD(By tercile, referent = 1, most deprived)	3	---	---	1.002 (0.953-1.053) p=0.944	---
	Univariate rural/urban classification (Rural referent)	4	---	---	---	0.976 (0.894-1.066) p=0.587
	Multivariate	5	0.969 (0.894-1.052) p=0.455	**0.906 (0.881-0.932) p<0.001**	1.018 (0.970-1.069) p=0.465	0.943 (0.865-1.028) p=0.181
Fever	Univariate Sex (Female Referent)	1	**0.915 (0.844-0.993) p=0.033**	---	---	---
	Univariate Age (Per Decade Above 18-24 Referent)	2	---	**0.911 (0.887-0.936) p<0.001**	---	---
	Univariate IMD(By tercile, referent = 1, most deprived)	3	---	---	1.007 (0.959-1.057) p=0.785	---
	Univariate rural/urban classification (Rural referent)	4	---	---	---	0.965 (0.885-1.052) p=0.419
	Multivariate	5	0.955 (0.882-1.035) p=0.264	**0.910 (0.885-0.935) p<0.001**	1.022 (0.975-1.072) p=0.365	0.934 (0.859-1.017) p=0.114
Persistent cough	Univariate Sex (Female Referent)	1	0.937 (0.865-1.014) p=0.105	---	---	---
	Univariate Age (Per Decade Above 18-24 Referent)	2	---	**0.909 (0.885-0.933) p<0.001**	---	---

	Univariate IMD(By tercile, referent = 1, most deprived)	3	---	---	0.984 (0.938-1.032) p=0.506	---
	Univariate rural/urban classification (Rural referent)	4	---	---	---	1.022 (0.939-1.111) p=0.615
	Multivariate	5	0.980 (0.906-1.059) p=0.604	**0.909 (0.885-0.934) p<0.001**	1.002 (0.956-1.049) p=0.94	0.986 (0.908-1.071) p=0.742
Loss of taste/smell	Univariate Sex (Female Referent)	1		**---**	---	---
	Univariate Age (Per Decade Above 18-24 Referent)	2	---	**0.910 (0.885-0.934) p<0.001**	---	---
	Univariate IMD(By tercile, referent = 1, most deprived)	3	---	**---**	1.002 (0.954-1.051) p=0.948	---
	Univariate rural/urban classification (Rural referent)	4	---	**---**	---	1.017 (0.934-1.108) p=0.698
	Multivariate	5	0.973 (0.899-1.053) p=0.501	**0.909 (0.885-0.935) p<0.001**	1.020 (0.972-1.069) p=0.422	0.984 (0.905-1.070) p=0.710
Altered taste/smell	Univariate Sex (Female Referent)	1	1.045 (0.968-1.129) p=0.258	---	---	---
	Univariate Age (Per Decade Above 18-24 Referent)	2	---	0.978 (0.952-1.004) p=0.091	---	---
	Univariate IMD(By tercile, referent = 1, most deprived)	3	---	---	0.985 (0.940-1.031) p=0.513	---
	Univariate rural/urban classification (Rural referent)	4	---	---	---	1.027 (0.947-1.114) p=0.524
	Multivariate	5	1.058 (0.979-1.144) p=0.153	0.976 (0.950-1.003) p=0.075	0.990 (0.945-1.037) p=0.657	1.017 (0.937-1.104) p=0.686

Table H: Knowing where to test

Logistic regression models for the association of not knowing where to test, including covariates of age, sex, education and where living, comparing those who answered “yes” and “no”. Further sensitivity analyses assuming missing responses to be “no” lowers the proportion of answered “yes” and attenuates p-values, while effect estimates are directionally consistent and statistically significant (e.g. education multivariate model per 4 years of education, OR=0.711 (0.619-0.813), p=8.84e-07). Modeling age as young (referent 18-54 years) vs old (55+), produced a similar estimate of the association of age (OR=2.1 (1.65, 2.69), p=2.12e-09) with not knowing where to test. Modeling education in 8-year categories starting prior to completion of secondary school (0-8, 9-16, and 17-24) was also similar OR=0.662, 0.528-0.830, p=3.65e-4). See Supplementary Figure 7 and 9 for additional sensitivity analyses and qualitative investigations.

Model	Number	(Intercept)	Sex (Female Referent)	Age in Decade (18-24 Years Referent)	Education in 4 Years (<=12 Years Referent)	Where Living (City Referent)
Univariate Sex	1	OR=0.65 (0.554-0.762) p=1.228e-07	OR=1.334 (1.064-1.675) p=1.277e-02	NA	NA	NA
Univariate Age	2	OR=0.327 (0.236-0.45) p=1.220e-11	NA	OR=1.207 (1.129-1.292) p=5.007e-08	NA	NA
Univariate Education	3	OR=1.069 (0.915-1.25) p=3.997e-01	NA	NA	OR=0.648 (0.56-0.747) p=3.281e-09	NA
Univariate Where Living	4	OR=0.653 (0.519-0.817) p=2.186e-04	NA	NA	NA	OR=1.201 (0.926-1.562) p=1.696e-01
Multivariate Education Adjusting for Age and Sex	5	OR=0.417 (0.288-0.601) p=3.101e-06	OR=1.218 (0.962-1.543) p=1.009e-01	OR=1.203 (1.119-1.294) p=5.456e-07	OR=0.655 (0.563-0.759) p=3.051e-08	NA
Education with Survey Weights	6	OR=1.099 (0.904-1.336) p=3.433e-01	NA	NA	OR=0.672 (0.564-0.799) p=7.842e-06	NA

Figure B: Qualitative demographic-knowledge relationships in UMD-CTIS.

Shown are the proportion (Wilson 95% confidence interval for binomial) reporting they did not know where to test (“yes” vs “yes” and “no”) across various demographic factors including: a) self-identified work type, b) where living (city,town, village/rural) vs technology used (none, smart phone, symptom tracking app). Missing responses are excluded in the primary analyses. c) Sensitivity analysis assuming missing responses are “no”, and are generally lower but with the same demographic patterns.

(a) Self-Identified Work Type: While confidence bounds are overlapping, work/profession of financial/insurance/real estate services, public administration, health, professional/scientific/technical activities were the lowest while transportation, tourism and construction were the highest.

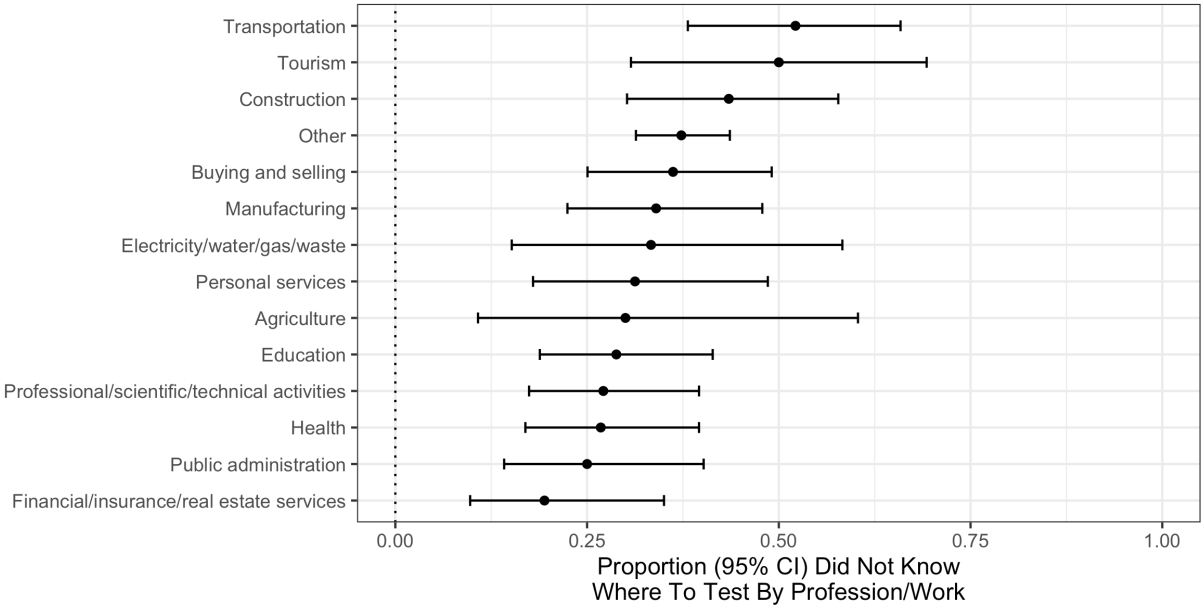

(b) Where living (city, town, village/rural) vs technology (none, smart phone, smart phone with tracking app). The proportion was lower among those using smart phone or symptom tracking app technology, regardless of whether living in a city, town or village/rural area.

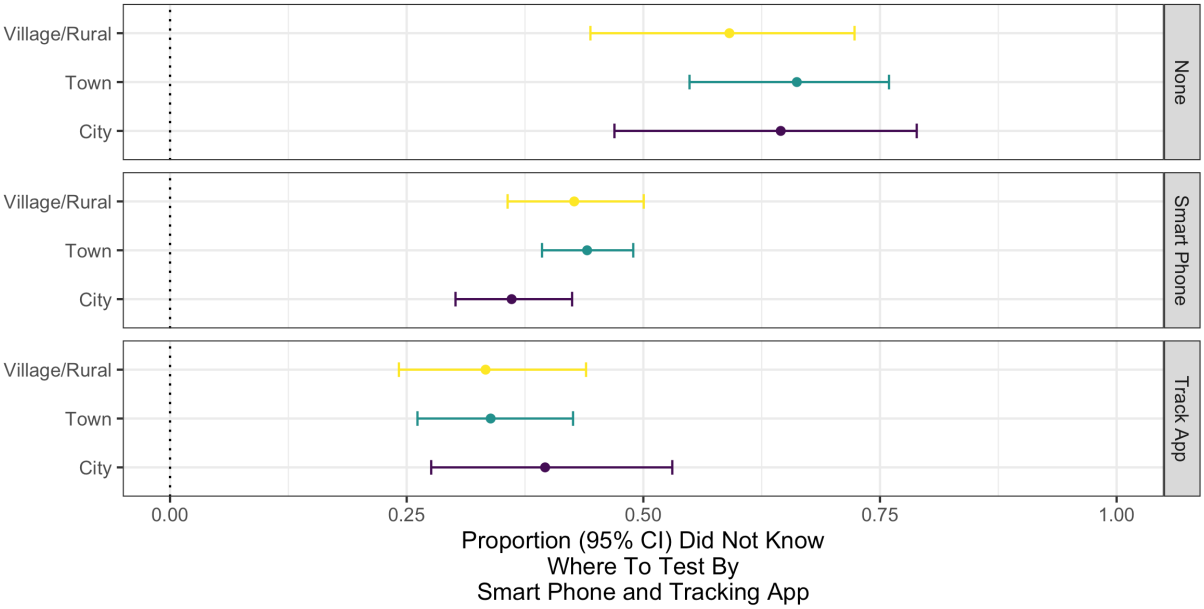

(f) Sensitivity analyses assuming missing responses are “no”, for where living (city, town, village/rural) vs technology (none, smart phone, smart phone with symptom tracking app). The absolute proportion not knowing where to go is lower when missing are assumed to be “no”, but

those using technology (smart phone, symptom tracking app) remain qualitatively lower than those not reporting using these.

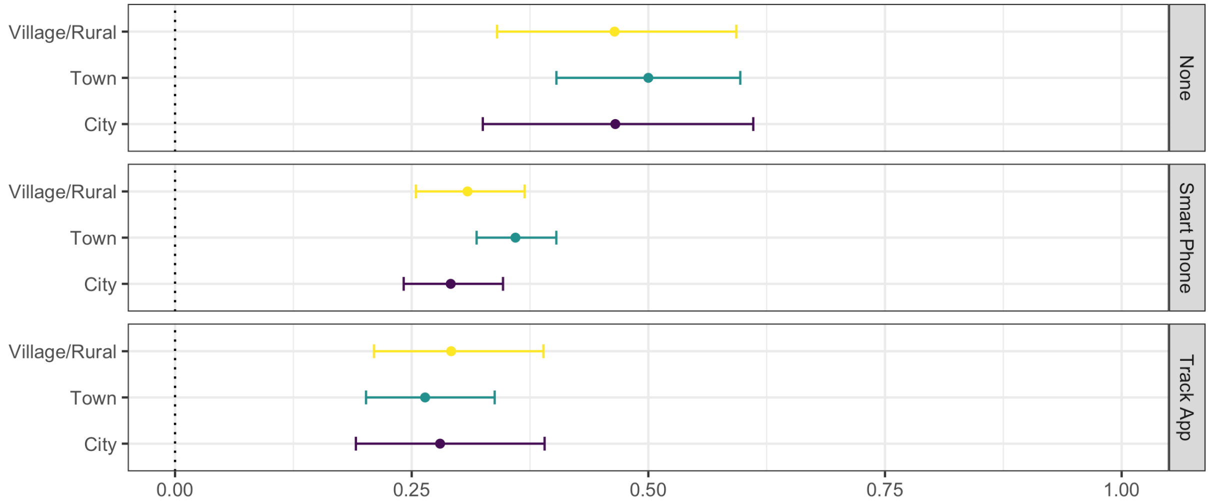

Proportion (95% CI) Did Not Know
Where To Test By
Smart Phone and Tracking App
Missing Responses Assumed No

Figure C: Participant locations for UMD-CTIS

Map of proportion of symptomatic, untested UMD-CTIS survey respondents who wanted to test but did not know where to test (a) proportion, (b) sensitivity analysis assuming missing responses were “no”, and (c) number of surveys with yes or no responses to the reason “I don't know where to go” when asked “Do any of the following reasons describe why you haven't been tested for coronavirus (COVID-19) in the last X days? [y/n]”. X is the self-reported duration of symptoms (up to 14 days) and (d) . (d) Geographic distribution of proportion of symptomatic ever tested during the study period and (e) early in the pandemic (Apr-Aug 2020). Not the orange (d) and yellow (e) color scales span the same percentage but have different minimum values. Early in the pandemic the testing gap was very similar across geographic areas. During the study period, the testing gap is closing in all geographic areas, with qualitatively better testing gains in the testing rates in some areas.

(a) Proportion “yes” vs “yes” plus “no”

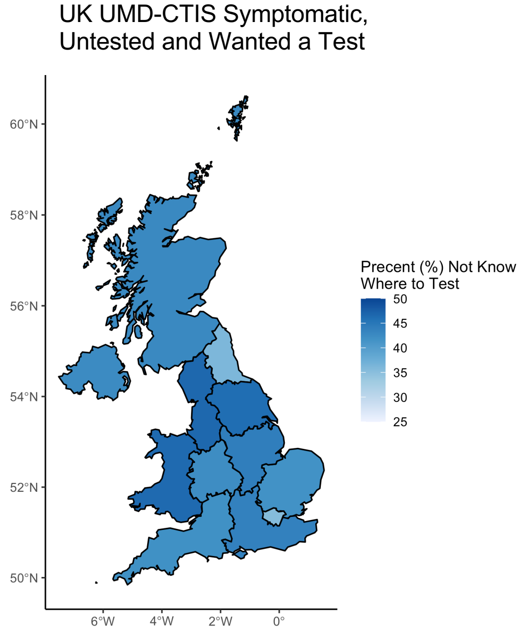

(b) Proportion “yes” vs “yes”, “no”, and missing

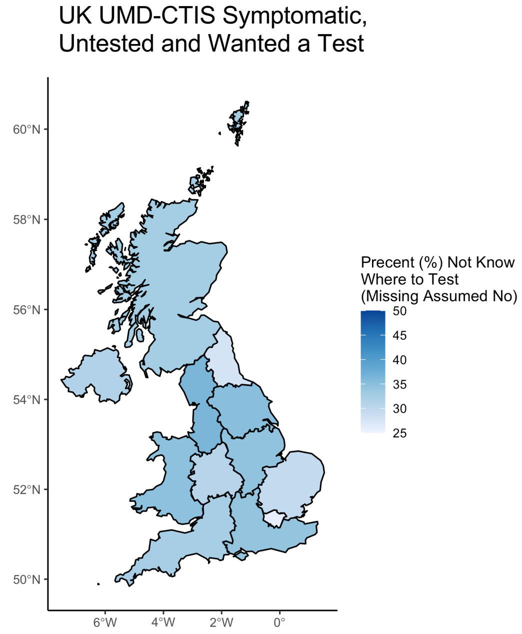

(c) Non-missing response count (yes plus no)

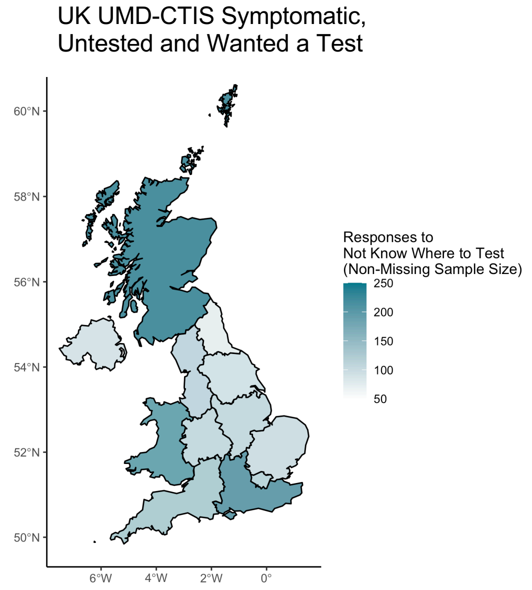

(d) Proportion ever tested (Dec 2020 to Feb 2021) (e) Proportion ever tested (Apr-Aug 2020)

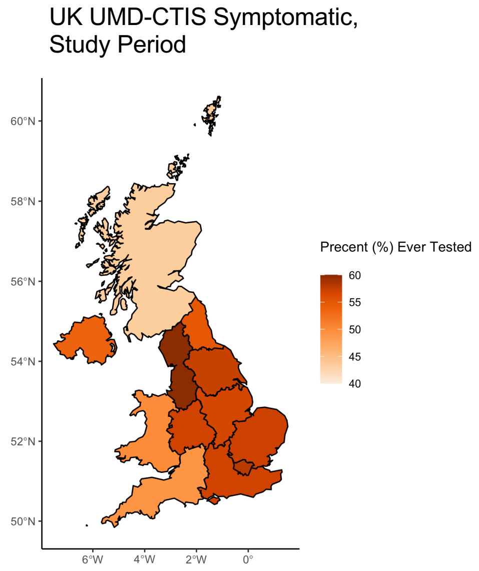

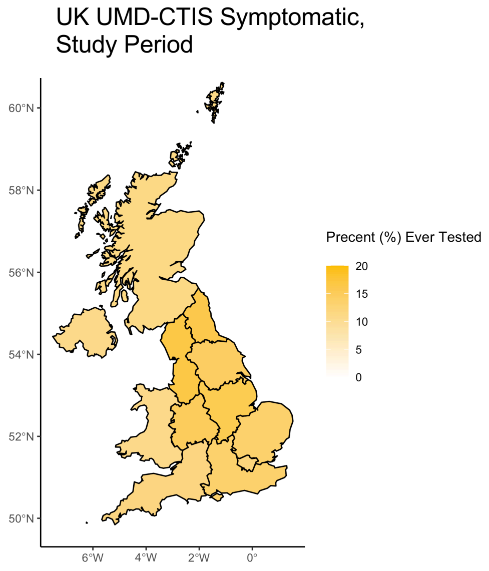

Contains National Statistics data © Crown copyright and database right [2021]. Contains Ordnance Survey (OS) data © Crown copyright [and database right] (2121). Office for National Statistics (2011). 2011 Census: boundary data (England and Wales) [data collection]. UK Data Service. SN:5819 UKBORDERS: Digitised Boundary Data, 1840- and Postcode Directories, 1980-.
http://discover.ukdataservice.ac.uk/catalogue/?sn=5819&type=Data%20catalogue,
Retrieved from http://census.ukdataservice.ac.uk/get-data/boundary-data.aspx.
Contains public sector information licensed under the Open Government Licence v3.
